# Risk Factors for Preterm Birth in Five Maternal and Child Health Hospitals in Beijing

**DOI:** 10.1371/journal.pone.0052780

**Published:** 2012-12-27

**Authors:** Yun-Ping Zhang, Xiao-Hong Liu, Su-Hong Gao, Jia-Mei Wang, Yue-Shan Gu, Jiu-Yue Zhang, Xia Zhou, Qing-Xia Li

**Affiliations:** 1 Department of Gynecology and Obstetrics, Maternal and Child Health Hospital, Haidian District, Beijing, China; 2 Department of Breast Surgery, Maternal and Child Health Hospital, Tongzhou District, Beijing, China; 3 Department of Anesthesiology, Maternal and Child Health Hospital, Shunyi District, Beijing, China; 4 Department of Pediatrics, Maternal and Child Health Hospital, Changping District, Beijing, China; 5 Department of Gynecology and Obstetrics, Maternal and Child Health Hospital, Daxing District, Beijing, China; University of Florida, United States of America

## Abstract

**Background:**

Preterm birth, the birth of an infant prior to 37 completed weeks of gestation, is the leading cause of perinatal morbidity and mortality. Preterm infants are at greater risk of respiratory, gastrointestinal and neurological diseases. Despite significant research in developed countries, little is known about the causes of preterm birth in many developing countries, especially China. This study investigates the association between sciodemographic data, obstetric risk factor, and preterm birth in five Maternal and Child Health hospitals in Beijing, China.

**Methods and Findings:**

A case-control study was conducted on 1391 women with preterm birth (case group) and 1391 women with term delivery (control group), who were interviewed within 48 hours of delivery. Sixteen potential factors were investigated and statistical analysis was performed by univariate analysis and logistic regression analysis. Univariate analysis showed that 14 of the 16 factors were associated with preterm birth. Inter-pregnancy interval and inherited diseases were not risk factors. Logistic regression analysis showed that obesity (odds ratio (OR) = 3.030, 95% confidence interval (CI) 1.166–7.869), stressful life events (OR = 5.535, 95%CI 2.315–13.231), sexual activity (OR = 1.674, 95%CI 1.279–2.191), placenta previa (OR 13.577, 95%CI 2.563–71.912), gestational diabetes mellitus (OR = 3.441, 95%CI1.694–6.991), hypertensive disorder complicating pregnancy (OR = 6.034, 95%CI = 3.401–10.704), history of preterm birth (OR = 20.888, 95%CI 2.519–173.218) and reproductive abnormalities (OR = 3.049, 95%CI 1.010–9.206) were independent risk factors. Women who lived in towns and cities (OR = 0.603, 95%CI 0.430–0.846), had a balanced diet (OR = 0.533, 95%CI 0.421–0.675) and had a record of prenatal care (OR = 0.261, 95%CI 0.134–0.510) were less likely to have preterm birth.

**Conclusions:**

Obesity, stressful life events, sexual activity, placenta previa, gestational diabetes mellitus, hypertensive disorder complicating pregnancy, history of preterm birth and reproductive abnormalities are independent risk factors to preterm birth. Identification of remedial factors may inform local health and education policy.

## Introduction

Preterm birth, the birth of an infant prior to 37 completed weeks of gestation, continues to be the leading cause of perinatal morbidity and mortality [1]. Infants born preterm are known to have a certain added risk of death, disease, disability, as well as longer-term motor, cognitive, visual, hearing, behavioral, social-emotional, health, and growth problems compared with normal-term infants [2], [3].

The underlying causes of preterm delivery are multiple and poorly understood.. It may include individual-level behavioral and psychosocial factors, neighborhood characteristics, environmental exposures, medical conditions, infertility treatments, biological factors, and genetics [4]. Many of these factors occur in combination.

Despite significant research in developed countries, there is little known about the causes of preterm birth in many developing countries. There are significant, persistent, and very disturbing racial, ethnic, and socioeconomic disparities in the rates of preterm birth. The study of the risk factors for preterm birth in China, especially in Beijing is rarely reported. As the capital of China, Beijing has special characteristics. The study on the effect mechanism of the public hygiene, demography, and social circumstance on the preterm birth in Beijing is of theoretical and practical significance for suppressing the preterm birth and thus for increasing the population quality of the capital. Therefore, a nationwide retrospective epidemiological study of preterm birth rates was performed in 2008 by Haidian Maternal and Child Health Hospital and the Medical Genetics Center of Peking University. A total of 55 hospitals in 15 cities were included in the study. There were nearly 300,000 live births, and over 15,000 preterm infants were identified, with a national preterm delivery rate of 5.63%. The rates varied markedly among different provinces as well as different levels of hospital. Although this was a nationwide study, risk factors for preterm birth could not be analyzed because of incomplete information. We thus decided to conduct a case-control, retrospective study in Beijing, China, that would compare pregnant women who had preterm birth with an appropriate control group to determine factors that might be associated with preterm birth.

## Methods

### Ethics Statement

This work was conducted according to the principles expressed in the Declaration of Helsinki, and approved by the Ethics Board, Haidian Maternal and Child Health Hospital, Beijing. Written informed consent was obtained from the participants at the time of their usual hospital care during the birth.

### Study design and subjects

This study was designed as a matched case-control study, at a ratio of 1∶1.

There are two kinds of hospitals capable to deliver a child in Beijing. They could be classified as Maternal and Child Health (MCH) hospitals and comprehensive hospitals. There are eighteen MCH hospitals in Beijing, with one MCH hospital in one District.This study was conducted between August 1, 2009 and August 31, 2010 at five MCH hospitals in Beijing, China, which are located in five districts: Haidian District, Tongzhou District, Shunyi District, Changping District and Daxing District. The skills of midwifery was almost same in the above five MCH hospitals. The delivery number in these five MCH hospitals was 32712 during the study period, accounted for 18.8% of the total delivery number in Beijing, and they located in different orientation of Beijing.

Cases were women who delivered before 37 completed weeks' gestation (20–36 weeks' gestation). Preterm delivery cases were identified by daily monitoring of all new deliveries at postpartum wards of participating hospitals. Controls were women who delivered at term (≥37 weeks' gestation). 1429 deliveries were preterm birth. The total preterm rate was 4.37%. The preterm rate of Haidian MCH, Tongzhou MCH, Shunyi MCH, Changping MCH and Daxing MCH were 5.32%(656/12333), 4.15%(326/7864), 3.93%(175/4458), 4.16%(147/3535) and 2.76%(125/4522) respectively. An eligible control, delivering immediately after a case, with similar age (±3 years), was approached and recruited for the study. Except incomplete data (people who could not be contacted with or would not participate in), there were 1391 preterm births and 1391 full-term deliveries analyzed, accounting for 97.34% (1391/1429) of all the preterm cases.

### Data collection

Standard operating procedures were elicited to all research personnel in the launching meeting before the formal study. The usage of standardized data collection instruments were explained in detail.

After obtaining informed consent, we asked the women to participate in a 20-minute personal interview in which trained research personnel used a standardized structured questionnaire to elicit information regarding maternal sociodemography, sex activity during pregnancy and medical history. Prenatal and delivery medical records were reviewed by trained research nurses who used a standardized abstraction form. Information abstracted from medical records included prepregnancy weight, height, pregnancy complications and condition of the newborn.

### Analytical variable specification

Gestational age was based on the last menstrual period (LMP) confirmed by sonographic examination prior to 20 weeks gestation. As for women who had a poor recall of LMP or the LMP and ultrasound date differed by more than 14 days, sonographic examination was used for measurement of gestational age. The total proportion of GW determined by ultrasound date was 20.6% (573/2782). Although the specific proportion of women who had a poor recall of LMP was not documented in this study, it was extremely low according to the daily clinical work.

Educational attainment was categorized as elementary school or less (≤6 years), middle school (6–12 years), university (13–18 years) or graduate and beyond (≥19 years of completed education).

BMI is defined as body weight divided by the square of height in kg/m^2^. BMI was classified into four categories [5]: underweight (<18.5), normal (18.5–24.9), overweight (25–29.9) and obese (≥30.0).

According to Chinese Nutrition Society, reasonable nutrition requires the heating percentage of three major nutrients were protein (eggs, milk, meat, poultry, fish) 10%–15%, fat (dairy products, oil) 20%–30% and carbohydrates (rice, sugar, sweets, bread, cakes) 55%–65%. Self-evaluation of pregnant women was acquired by personal interview.

Inter-pregnancy interval was defined as that interval between the termination of one pregnancy and the conception of another and was categorized as four groups: 6 months or less, 7–12 months, 13–24 months and >24 months.

All pregnancies were screened for gestational diabetes mellitus (GDM) between 24 and 28 weeks of gestation. The screening test is a 50 g glucose load, given orally at any time of the day, with plasma glucose measured 1 h later. If plasma glucose is 7.8 mmol/L or greater, an oral glucose tolerance test (OGTT) is requested. The OGTT comprises plasma glucose determinations, the first while fasting and subsequently at 1, 2 and 3 h after ingesting a 75 g glucose load. GDM is diagnosed when any two of these plasma glucose values are equal or greater to the following values: fasting ≥5.6 mmol/L, 1 h ≥10.3 mmol/L, 2 h ≥8.6 mmol/L, 3 h ≥6.7 mmol/L).

Hypertensive disorder complicating pregnancy was defined according to the textbook of Obstetrics and Gynecology [6] as a recorded blood pressure ≥140/90 mmHg on more than two occasions more than 6 h apart.

### Statistical analysis

The data were analyzed using the Statistical Package for the Social Sciences (SPSS) version 11.5. Variables with more than 10% missing data were excluded from analyses. A paired-sample Student *t*-test was used for comparison of continuous variables. Comparison of proportions was performed by the McNemar-Bowker test as appropriate. Statistical significance was achieved when at *P*<0.05. Conditional logistic regression analysis was used to determine independent risk factors for preterm delivery. Cox regression analysis [7] was employed, with backward selection to identify variables for the final model. Odds ratios (ORs) were determined. Missing data was handled in the multivariable analysis by omitting participants with any missing data. All reported *P* values are 2 tailed, and confidence intervals(CIs) were calculated at the 95% level.

## Results

The mean maternal age of the participants was (28.57±4.55) years for the preterm group and (28.68±4.14) years for the full-term group, and the difference was not significant (*t* = −1.898, *P* = 0.058). The gestation weeks of the preterm group were in Table 1.

**Table 1:** The distribution of gestation week(GW) of preterm birth.

Hospitals	Sample number	Gestation week	20∼27^+6^GW		28∼31^+6^ GW		32∼33^+6^ GW		34∼36^+6^ GW	
			n	percentage (%)	n	percentage (%)	n	percentage (%)	n	percentage (%)
Haidian MCH	651	34.46±3.009	34	5.2	23	3.5	52	8.0	542	83.3
Tongzhou MCH	322	33.98±3.578	24	7.5	18	5.6	21	6.5	259	80.4
Shunyi MCH	174	34.87±1.856	3	1.7	6	3.4	15	8.6	150	86.2
Changping MCH	140	34.72±1.714	0	0	10	7.1	14	10.0	116	82.9
Daxing MCH	104	34.40±3.139	7	6.7	4	3.8	5	4.8	88	84.6
Total	1391	34.42±2.953	68	4.9	61	4.4	107	7.7	1155	83.0

### Univariate analysis

As shown in Table 2, univariate analysis indicated that preterm birth occurred more frequently among women who were unmarried (OR = 6.600, 95%CI 2.577–16.906), and had education to elementary school or less (OR = 1.441, 95% CI 1.032–2.012). Living in a town or city was associated with a smaller risk of preterm birth than living in a village.

**Table 2:** Characteristics of participants in the preterm birth group and control group.

	Preterm group		Control group		*P*	OR	95%CI
	*n*	%	*n*	%			
Marital status							
Married	1356	97.5	1384	99.5	0.000	1.000	
Unmarried	35	2.5	7	0.5		6.600	2.577–16.906
Education							
Elementary school or less	610	44.1	533	38.7	0.032	1.441	1.032–2.012
Middle school	558	40.4	585	42.5	0.776	1.048	0.760–1.445
University	125	9.1	159	11.5	0.216	0.774	0.515–1.162
Graduate	88	6.4	101	7.3		1.000	
Community							
Town/city	961	70.6	1060	77.3	0.000	0.537	0.422–0.683
Village	400	29.4	312	22.7		1.000	

OR: odds ratio; CI: confidence interval.

Table 3 shows that obesity before pregnancy (BMI≥30.0 kg/m^2^) was associated with an increased risk of preterm birth. In this study, stressful life events for the participants included hospitalization, surgery or death of family members, and family conflict. Exposure to life events (OR = 4.400, 95% CI 2.116–8.743), house or office decorating (OR = 1.960, 95% CI 1.211–3.173), as well as having sex during pregnancy (OR = 1.451, 95% CI 1.189∼1.771) were all risk factors for preterm birth. Fewer women in the preterm group (59.2%) had a balanced diet compared with the control group (69.5%). A balanced diet was associated with a decreased risk of preterm birth (OR = 0.560, 95% CI 0.466–0.672). Women with a record of prenatal care were less likely to have preterm birth (OR = 0.227, 95% CI 0.147–0.351).

**Table 3:** Maternal health during pregnancy and risk of preterm birth.

	Preterm group		Control group		*P*	OR	95%CI
	*n*	%	*n*	%			
Pre-pregnancy BMI (kg/m^2)^							
<18.5	180	13.9	202	15.0	0.956	0.994	0.791–1.248
18.5–24.9	858	66.4	928	68.8		1.000	
25–29.9	227	17.6	206	15.3	0.124	1.192	0.953–1.492
≥30.0	28	2.0	12	0.9	0.005	2.946	1.377–6.302
Balanced diet							
No	557	40.8	419	30.5		1.000	
Yes	809	59.2	956	69.5	0.000	0.560	0.466–0.672
Stressful life event							
No	1288	96.6	1352	99.2		1.000	
Yes	46	3.4	11	0.8	0.000	4.400	2.116–8.743
Home or office decoration							
No	1315	94.5	1339	96.3		1.000	
Yes	76	5.5	52	3.7	0.006	1.960	1.211–3.173
Sex							
No	695	50.0	771	55.4		1.000	
Yes	696	50.0	620	44.6	0.000	1.451	1.189–1.771
Prenatal care							
No	116	8.3	31	2.2		1.000	
Yes	1275	91.7	1360	97.8	0.000	0.227	0.147–0.351

Table 4 presents the relationship of pregnancy complications with preterm birth. Placenta previa (OR = 9.50, 95% CI 2.213–40.784), GDM (OR = 3.222, 95% CI 1.899–5.468), hypertensive disorder complicating pregnancy (OR = 3.972, 95% CI 2.756–5.725) were all associated with increased risk of preterm birth.

**Table 4:** Pregnancy complications and risk of preterm birth.

	Preterm group		Control group		*P*	OR	95%CI
	*n*	*%*	*n*	*%*			
Placenta previa							
No	1287	98.5	1315	99.8		1.000	
Yes	19	1.5	3	0.2	0.002	9.500	2.213–40.784
Gestational Diabetes Mellitus							
No	1323	95.6	1353	98.5		1.000	
Yes	61	4.4	21	1.5	0.000	3.222	1.899–5.468
Hypertensive disorder complicating pregnancy							
No	1242	89.3	1349	97.0		1.000	
Yes	149	10.7	42	3.0	0.000	3.972	2.756–5.725

The associations between obstetric history, family history and preterm birth are shown in Table 5. Differences in risk between the four inter-pregnancy interval categories and preterm birth were not statistically significant. The occurrence of preterm labor had a significant positive correlation with the recurrence of preterm delivery (OR = 9.667, 95% CI 2.945–31.732). Reproductive abnormalities in this study included longitudinal vaginal septum, uterus bicornis, saddle form uterus and uterus septus, and were related to preterm birth. Family genetic diseases in the study included thalassemia, fabism, achromatopsia, hypochromatopsia, thrombocytopenia, Down's syndrome, and hyperdactylia. Numbers were too small to investigate the impact of individual disorders and it was not shown that they were associated with the increased risk of preterm birth.

**Table 5:** Obstetric history, family history and risk of preterm birth.

	Preterm group		Control group		*P*	OR	95%CI
	*n*	*%*	*n*	*%*			
Inter-pregnancy intervals							
≤6 months	65	10.7	78	11.7		1.000	
7–12 months	134	22.1	136	20.3	0.605	1.184	0.624–2.248
13–24 months	137	22.6	157	23.5	0.459	1.256	0.687–2.296
>24 months	269	44.5	298	44.5	0.134	1.564	0.871–2.809
History of preterm birth							
No	1362	97.9	1388	99.8		1.000	
Yes	29	2.1	3	0.2	0.000	9.667	2.945–31.732
Reproductive abnormality							
No	1355	98.3	1372	99.6		1.000	
Yes	23	1.7	6	0.4	0.003	3.333	1.561–9.414
Family genetic diseases							
No	1384	99.6	1385	99.9		1.000	
Yes	6	0.4	2	0.1	0.178	3.000	0.606–14.864

### Multivariate analysis

After adjusting for confounding factors, the results of multiple logistic regression analysis are presented in Table 6. The significant risk factors for preterm delivery were obesity (OR = 3.030, 95%CI 1.166–7.869), stressful life events (OR = 5.535, 95%CI 2.315–13.231), having sex during pregnancy (OR = 1.674, 95%CI 1.279–2.191), placenta previa (OR = 13.577, 95%CI 2.563–71.912), GDM (OR = 3.441, 95%CI 1.694–6.991), hypertensive disorder complicating pregnancy (OR = 6.034, 95%CI 3.401–10.704), history of preterm birth (OR = 20.888, 95%CI 2.519–173.218) and a reproductive abnormality (OR = 3.049, 95%CI 1.010–9.206). However, women who lived in towns and cities (OR = 0.603, 95%CI 0.430–0.846), had a balanced diet (OR = 0.533, 95%CI 0.421–0.675) and had prenatal care (OR = 0.261, 95%CI 0.134–0.510) were less likely to have preterm birth. Less education was associated with an increased risk of preterm birth in the unadjusted analysis, but this finding was not statistically significant after accounting for confounding risk factors for preterm birth in this group of women.

**Table 6:** Risk factors for preterm birth.

Related factors	β	Waldχ^2^	*P*	OR	95%CI
Unmarried	1.074	2.504	0.114	2.926	0.774–11.059
Community	−0.505	8.590	0.003	0.603	0.430–0.846
Pre-pregnancy BMI (kg/m^2^)		8.175	0.043		
<18.5	0.087	0.360	0.549	1.091	0.821–1.448
25–29.9	0.273	3.397	0.065	1.313	0.983–1.755
≥30.0	1.108	5.181	0.023	3.030	1.166–7.869
Balanced diet	−0.629	27.227	0.000	0.533	0.421–0.675
Prenatal care	−1.343	15.415	0.000	0.261	0.134–0.510
Stressful life events	1.711	14.807	0.000	5.535	2.315–13.231
Sex	0.515	14.068	0.000	1.674	1.279–2.191
Placenta previa	2.608	9.404	0.002	13.577	2.563–71.912
Home/office decorating	0.578	2.370	0.124	1.7783	0.854–3.722
Gestational diabetes mellitus	1.236	11.679	0.001	3.441	1.694–6.991
Hypertensive disorder complicating pregnancy	1.797	37.771	0.000	6.034	3.401–10.704
History of preterm birth	3.039	7.929	0.005	20.888	2.519–173.218
Reproductive abnormality	1.115	3.911	0.048	3.049	1.010–9.206

## Discussion

A major strength of this study was its large sample size with matched case-control design and identification of risk factors was based on 1391 women with their pregnancies complicated by preterm birth. The strength of our study also lies in the use of patient interviews and medical record data rather than birth certificate data. Thus, the association between more potential risk factor and the presence of preterm delivery was investigated. It should be noted that estimates generated by logistic regression analysis of data in this retrospective study should be interpreted with caution, especially as the numbers for some risk factors were small and the confidence intervals were wide.

We concur with prior studies that GDM [8], hypertensive disorder complicating pregnancy [9], history of preterm birth [10], stressful life events [11], and reproductive abnormalities [9] are associated with an increased risk of preterm birth. The OR values varied from those in previous studies, and may be related to the different geographical area. Previa placenta can cause fetal distress due to overmuch bleeding, even hypoxia. It is always decided to terminate pregnancy to save the life of the pregnant woman and/or the fetus, resulting in a corresponding increase in indicated preterm delivery.

There is certain genetic basis for preterm birth [12]. Both the mother's genotype [13] and fetal genotypes are associated with preterm birth, and mother's genotype may play a decisive role. Norway 1967–1976 birth data [14] show that, the probability of preterm delivery for women with once history of preterm delivery is 14.3%, and it increases to 28.1% for women with twice history of preterm birth. To date, most published studies on the genetics of preterm birth have examined only one or a few genes in a given study sample. New tools for high-throughput genotyping, coupled with very-large-scale population-based studies that use sensitive biomarkers, comprehensive exposure assessment, and advanced biotechnology and analytical strategies, are needed to unravel the complex environmental and genetic factors, and gene-gene, and gene-environment interactions responsible for preterm birth. Understanding these factors and their interactions could lead to major improvements in the diagnosis, prevention, and treatment of preterm birth.

After adjustment for potential confounders, multivariate logistic regression analysis indicated that women with prenatal care were less likely to have preterm birth (OR = 0.252, 95%CI 0.130–0.489), which is compatible with a previous study [15] that women without prenatal care were more likely to have preterm labor (OR = 3.1, 95%CI 1.4–6.8). Our analysis on the role of prenatal care in preterm birth supports the conclusion that the prevention of preterm births is linked to the availability of and access to prenatal care [16].

The present study showed that living in a town or city was associated with a lower risk of preterm birth, even after adjusting for other factors. Although many efforts have been made in Beijing, there are a wide medical and health care gap and a wide income gap between urban and rural areas. This concurs with studies that showed that living conditions may directly increase the occurrence of preterm birth through neuroendocrine and immune pathways that affect susceptibility to infection and hypertension [17]–[19]. Other environmental effects may indirectly influence maternal behavior such as short inter-pregnancy interval, and access to prenatal care [20], [21]. These risk factors are intimately linked with the household income and the level of medical and health care. This is further evidence that no prenatal care and stressful life events are related to preterm birth, which is also shown in our study.

Life events are major events that individuals experience, such as divorce, a death in the family, illness, or injury. It makes people suffered from stress, threaten the homeostasis. In this study, stressful life events for the participants included hospitalization, surgery or death of family members, and family conflict. Nedra Whitehead and colleagues [22] studied the relation between number of stressful life events and preterm delivery in 11 US states. The result showed that the risk of preterm delivery among multiparas who gave birth in 1990–1993 increased 7% for each event over five they experienced, among primiparas who gave birth in 1994–1995, the risk increased 5% for each event over two.

The potential for adverse effects of sexual activity, particularly intercourse, during pregnancy has been of concern for some time due to the potential for direct effects of semen on initiating preterm labor, alteration of vaginal microflora, or other hypothesized pathways leading to preterm birth. Engaging in sexual activity was associated with an increased risk of preterm birth in our study, but other studies reported an absence of increased risk and even a notably diminished risk of preterm birth [23]. Maybe pregnant women are relatively more sensitive to items related to sexual activity, as we've realized in the pilot study. So, the two items were designed as self-answered and 99.3% (2762/2782) of the subjects responded, with 47.6% (1316/2762) selected “have had sexual activity during pregnancy”, among which only 32.1% (422/1316) responded to the second item “which trimester”. Moreover, the second item was mutli-optional, that was any one trimester or any two trimester or all three trimesters were all acceptable. So, sexual activity during pregnancy should be adjusted by timing or trimester in further study.

Although the definition of balanced diet is rough and the information maybe rough, a balanced diet was associated with a decreased risk of preterm birth in this study, which provided further support for obesity as a risk factor for preterm birth. A previous study showed that obesity was an independent risk factor for late preterm birth [24]. A similar result was found in our work. However, one study found that obese women were at a markedly decreased risk of spontaneous preterm birth [25]. Different standards of weight gain during pregnancy have been set for different pre-pregnancy BMI, so the relationship of different weight gains during pregnancy stratified by pre-pregnancy BMI with preterm birth needs to be studied further. So far, no study of Chinese-specific weight gain during pregnancy stratified by pre-pregnancy BMI has been reported.

Inter-pregnancy interval was not associated with preterm birth in our study, which is not consistent with results from a previous study [26]. This probably result from a different cutoff value for the inter-pregnancy interval on transfer from a continuous variable to a discrete variable and requires further analysis.

Given the complexity of the influencing factors of preterm birth, there are some limitations in this study. Firstly, our study may be limited by recall bias since mothers were interviewed after delivery, for example, sexual activity during pregnancy. Secondly, our study did not classify preterm birth by sub-categories. Preterm birth is typically defined as a delivery or birth at a gestational age less than 37 weeks. Other commonly used subcategories of preterm birth have been established. They are extremely preterm (birth at ≤28 weeks of gestation), very preterm (birth at <33 weeks of gestation) and moderately preterm (birth at 33 to 36 weeks of gestation). It could also be classified into three separate subgroups according to clinical presentation: births occurring after spontaneous premature labor; births related to premature contractions; and spontaneous rupture of the membranes and indicated delivery of a premature infant for the benefit of either the infant or the mother. The influencing factors of the different sub-categories of preterm birth needs to be further studied. Lastly, because the five hospitals were not the terminal comprehensive health center, so the pregnancy women with critical disease were referred to the tertiary comprehensive health center, thus the pregnancy complications mainly included hypertensive disorder complicating pregnancy and gestational diabetes mellitus. The representative of the result is subject to certain restriction.

In conclusion, this study indicated that obesity, stressful life events, sexual activity, placenta previa, GDM, hypertensive disorder complicating pregnancy, history of preterm birth and reproductive abnormalities are independent risk factors for preterm birth. Although advances in high-risk obstetric and neonatal care have resulted in improved survival of infants born preterm, many studies have documented the prevalence of a broad range of neurodevelopmental impairments in preterm survivors. The spectrum of neurodevelopmental disabilities includes cerebral palsy, mental retardation, visual and hearing impairments, and more subtle disorders of central nervous system function. So, develop new premature predictors to identify high-risk groups and take effective intervention to improve birth outcomes is still an important direction of the future.
